# Nutritional potential of wild sorghum: Grain quality of Sudanese wild sorghum genotypes (*Sorghum bicolor* L. Moench)

**DOI:** 10.1002/fsn3.1002

**Published:** 2019-03-25

**Authors:** Tilal Sayed Abdelhalim, Nasrein Mohamed Kamal, Amro B. Hassan

**Affiliations:** ^1^ White Nile Research Station Agricultural Research Corporation Kosti Sudan; ^2^ Biotechnology and Biosafety Research Center Agricultural Research Corporation Shambat, Khartoum North Sudan; ^3^ Arid Land Research Center Tottori University Tottori Japan; ^4^ Environment and Natural Resource and Desertification Research Institute (ENDRI) National Center for Research Khartoum Sudan

**Keywords:** biofortification, genotypes, grains, micronutrients, wild sorghums

## Abstract

In the last decades, deficiency of macro‐ and micronutrients was considered as a serious problem associated with the increase in the human population. To meet the increased demand for food consumption, the wild relative plant might serve as an important source of new genetic material for increasing macro‐ and micronutrients. To investigate this, the variations in protein content, in vitro protein digestibility, tannin content, phytic acid content, total polyphenol content, and total and bioavailability of minerals were studied in grains of ten wild sorghums and two released sorghum cultivars. The results showed significant differences (*p* ≤ 0.05) in all quality tests among the genotypes. The highest percentage of total protein contents and in vitro protein digestibility were encountered in the grains of PQ‐434 (14.6%) and the released cultivar AG8 (49.8%), respectively, while the highest concentrations of total and bioavailable iron were found in the grains of Almahkara (3.17 mg/100 g) and Abusabiba (92.8 mg/100 g), respectively. The grains of wild sorghum genotype Adar Umbatikh grains were found to possess higher total zinc contents. The PCA identified only five components of eigenvalues greater than one and cumulatively accounted for 88% of the total variation. It could be concluded that Almahkara and PQ‐434 could be used as potential sources for iron and protein sorghum biofortification, respectively. Results from this study might be used in the development of new value‐added products from wild sorghum grains by‐products.

## 1 INTRODUCTION

Sorghum (*Sorghum bicolor* L. Moench, 2n = 2x = 20) is a globally important food security crop, particularly in arid and semiarid environments. It is the main staple food for about 300 million people who live in the semiarid tropics (Orr, Gierend, & Swamikannu, 2016). The crop is a C4 species with high photosynthetic capacity and inherent high biomass yield potential. Its high levels of tolerance to drought and high temperature, and adaptation to inherent low soil fertility make the crop progressively more relevant for food security in view of climate change. In addition, it is a crop for poor communities.

In Sudan, sorghum is a multipurpose crop and cultivated in almost all regions by subsistence farmers for wide use. It has been used to prepare different kinds of traditional food such as leavened bread “kisra,” porridge “Asida,” and animal feed and to prepare local beverages “Abraih” (Dirar, 1994). Sorghum grains are also considered as one of the major components of livestock and poultry feed. Further, the stalk is also used as animal feed and for house and fence construction. The grain is characterized by its high starch, protein, micronutrients, and crude fiber but low in fat (Kumar et al., 2015). It has been reported that majority of the Sudanese low‐income people in rural areas, where both physical and economic access to nutrient‐rich foods are limited, depend on sorghum for their dietary energy and micronutrient requirement (Yonos, 2001).

Micronutrient malnutrition causes blindness and anemia (even death) in more than half of the world's population, especially among women of reproductive age, pregnant and lactating women, and preschool children (Bailey, West, & Black, 2015; Graham, Welch, & Bouis, 2001). Recently, the UN Millennium Development Goal progress report for Sudan revealed that 31 percent of children under the age of five in Sudan are moderately or severely underweight (Musa, Musa, Ali, & Mustafa, 2014). In the study of Abdelrahim et al. (2009), anemia during pregnancy was found to be a major health problem in Sudan, affecting around half of the women in some areas. It is a risk factor for maternal and perinatal morbidity and mortality. Therefore, enhancement of grain nutrients (biofortification), either agronomically (application of mineral fertilizers) or genetically (breeding), is viewed the most promising and cost‐effective approach to combat malnutrition and related health problems (Cakmak, 2007).

Biofortification of sorghum grain through genetic strategies is a powerful approach for changing the nutrient balance in the human diet on a large scale. In the context of developing fortified foods for low‐income consumers, different studies have revealed a narrow range of genetic diversity for sorghum grain micronutrient contents, with Fe and Zn contents (Ashok Kumar, Anuradha, & Ramaiah, 2013; Kumar et al., 2015). The narrow genetic diversity for grain minerals in modern cultivars could be attributed to eroded genetic diversity by domestication and breeding processes. Therefore, efforts must be devoted to identify valuable alleles which have been “left behind” in the wild ancestors of crop plants and to reintroduce them into cultivated crops (Tanksley & McCouch, 1997). Interestingly, wild wheat genotypes were found to possess high concentrations of micronutrients in grains compared to the domesticated cultivars (Monasterio & Graham, 2000). Furthermore, Peleg et al. (2008) showed large genetic variation among 22 wild emmer wheat accessions for Zn, Fe, and protein concentrations. The authors suggested that crop wild relatives might serve as an important source for increasing micronutrient concentration (Peleg et al., 2008). Hence, exploiting the genetic variation of wild sorghum collections in Sudan for their grain minerals and protein contents might attract special interest for several reasons. Since Sudan is within the geographical range where sorghum is postulated to be domesticated for the first time (Assar et al., 2005), where the largest genetic variation exists. To our best knowledge, no reports on minerals and protein contents have been published for wild and weedy sorghum. The overall objective of this study was, therefore, to explore the potential of wild sorghums as sources for improving the nutritional quality of grain. To do so, ten Sudanese wild sorghum genotypes and two released cultivars were screened in this study for antinutritional factor contents, crude and in vitro protein digestibility, and eventually total and bioavailable calcium, phosphorus, iron, and zinc contents.

## 2 MATERIALS AND METHODS

### 2.1 Samples collection and Experimental setup

Ten genotypes of wild/ weedy species of sorghum were collected, from the border areas between Sudan, Eritrea, and Ethiopia, a place where sorghum is postulated to be domesticated for, the first time, 5,000 years. The wild genotypes were given names according to their areas of the collection, classified as *Sorghum bicolor* var verticiliflorum and *Sorghum bicolor* var arundnacieum. The released sorghum cultivars, namely Tabat and AG8, were obtained from the sorghum breeding program at the Agricultural Research Corporation, Wad Medani, Sudan. To affirm the homogeneity, the wild sorghum genotypes were cultivated for three consecutive successive cycles using a single seed descent method of plant breeding to affirm the homogeneity. The field experiment was conducted at the White Nile Research Farm, White Nile State, Sudan, during 2014 and 2015 cropping seasons: Latitude 13° 10ʹ N, longitude 32° 40' E, and altitude 410 meters above sea level. The climate of the locality is semiarid with a cool dry winter and a hot summer. The rainy season extends from July to October with peak monthly rainfall in August. The mean annual rainfall is around 330 mm. The soil of the farm is characterized by high clay content, heavily cracking vertisols. The genotypes were sown in a two‐row plot of four meters length with the plant‐to‐plant distance of 20 cm and row–row spacing of 80 cm. The treatments were arranged in a randomized complete block design (RCBD) with three replications. N fertilizer in form of urea was applied uniformly at a rate of 43 kg N/ha once time. Panicles were harvested and threshed from cultivars and wild genotypes at physiological maturity. Due to the frequent shattering of spikes in various wild genotypes, the collection of mature spikes had to be done repeatedly at different intervals over 3–14 days. Due to tough glumes and hard threshing in wild species, the grains had to be taken out manually.

### 2.2 Sample preparation

Grains of the wild sorghum and released sorghum genotypes used in this study were carefully cleaned and freed from foreign materials. The whole grain of the samples was milled in the laboratory miller (Retsch, Germany) using 0.4‐mm sieve to produce fine flour. The milled samples were then packaged in a polyethylene bag and kept into a desiccator at room temperature during the analysis. All chemicals used in this study were of analytical grade.

### 2.3 Determination of crude protein and in vitro protein digestibility

The crude protein was determined following Kjeldahl method described by AOAC (2000). The in vitro protein digestibility (IVPD) was determined by the procedure of Maliwal (1983) cited from Monjula and John (1991). A known weight of the sample containing 16 mg nitrogen was taken in triplicate and digested with 1 mg pepsin in 15 ml of 0.1 M HCl at 37°C for 2 hr. The reaction was stopped by addition of 15 ml of 10% trichloroacetic acid (TCA). The mixture was then filtered quantitatively through Whatman No. 1 filter paper. The TCA‐soluble fraction was assayed for nitrogen using the micro‐Kjeldahl method (AOAC, 2000).

$$\text{in vitro Protein digestibility}\;\%\;=\frac{\text{digestible protein}}{\text{total protein}}\times 100.$$

### 2.4 Determination of total mineral

Mineral content of the grains was determined following the method of Chapman and Pratt (1982). Each sample was burnt in a muffle furnace at 550°C and then placed in a sand bath for 10 min after the addition of 5 ml of 5N HCl. The solution was carefully filtered in a 100‐ml volumetric flask, and finally, distilled water was added to make up to mark. The extracts were stored in bottles for further analysis. Fe and Zn contents were determined using atomic absorption spectrophotometer. Phosphorus was determined by spectrophotometric method using molybdovanadate, and the absorbance was measured at 730 nm.

### 2.5 Determination of minerals’ HCl extractability (in vitro bioavailability)

Minerals in the samples were extracted by the method described by Chauhan and Mahjan (1988). About 1.0 g of the sample was shaken with 10 ml of 0.03 M HCl for 3 hr at 37°C and then filtered. The clear extract obtained was oven‐dried at 60°C and then acid‐digested according to Chapman and Pratt (1982). HCl extractability (%) of the mineral was determined as follows:

HCl extractability%=(mineral extractable in0.03N HCl÷total mineral)×100.

### 2.6 Determination of tannin content

Tannin content of the samples was determined using the modified spectrophotometric vanillin–HCl method according to Price, Socoyoc, and Butter (1978) by using 200 mg sample. A standard curve was prepared and results were expressed as catechin equivalents, that is, amount of catechin (mg/g) which gives a color intensity equivalent to that given by tannins after correcting for blank.

### 2.7 Determination of total polyphenol

Total polyphenols were determined according to Prussian Blue spectrophotometric method (Price et al., 1978). Sixty milligrams of the ground sample was shaken manually for 1 min in 3.0 ml methanol. The mixture was filtered. The filtrate was mixed with 50 ml distilled water and analyzed within an hour. About 3.0 ml of 0.1 M FeCl_3_ in 0.1 M HCl was added to 1.0 ml of the filtrate followed immediately by timed addition of 3.0 ml of freshly prepared K_3_Fe(CN)_6_. The absorbance was monitored on a spectrophotometer at 720 nm after 10 min from the addition of 3.0 ml of 0.1 M FeCl_3_ and 3.0 ml of 0.008 M K_3_Fe(CN)_6_.

### 2.8 Determination of phytic acid content

Determination of phytic acid content was carried out by the method described by Wheeler and Ferrel (1971) by using 2.0 g dried sample. A standard curve was prepared, and results were expressed as Fe (NO_3_)_3_ equivalent. Phytate phosphorus was calculated from the standard curve assuming a 4:6 iron‐to‐phosphorus molar ratio.

### 2.9 Statistical analysis

Data were tested for normality of distribution and homogeneity of variances using Shapiro–Wilk and Levene's tests, respectively. Differences between treatments were measured by ANOVA, using STAR—Statistical Tool for Agricultural Research (version 1.0; http://bbi.irri.org/). Multiple comparisons were analyzed using Tukey range test. Broad‐sense heritability (H2) of the mineral elements and protein was estimated from the analysis of variance following Nyquist and Baker (1991). The formula used was H2 = VG/VP. PCA was performed using STAR software (STAR, version 1.4. International Rice Research Institute (IRRI), Los Baños, The Philippines; http://bbi.irri.org/products). Cluster analysis based on Euclidean distance was performed in order to employ the associations among the traits for assigning the germplasm lines and varieties to different clusters.

## 3 RESULTS AND DISCUSSION

### 3.1 Crude protein content and in vitro protein digestibility (IVPD) of the wild sorghum genotypes and released sorghum cultivars

**Figure 1:**
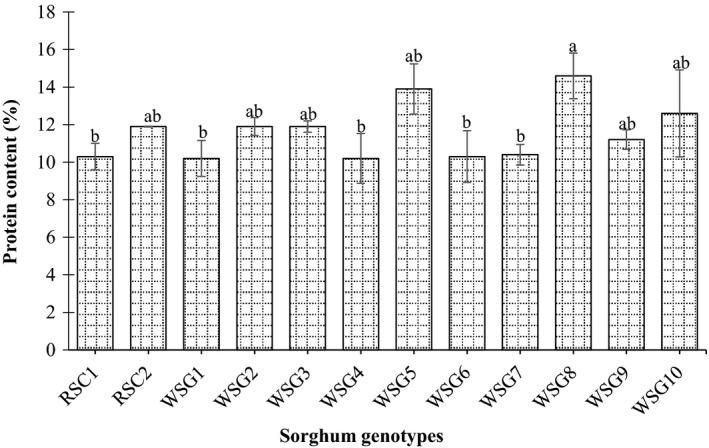
Protein content (%) of released sorghum cultivar and wild sorghum genotypes grains. Values are means (±*SD*) of triplicate samples. Values followed by the same letter are not significantly different (*p* < 0.05) as assessed by LSD. RSC1 (AG8); RSC2 (Tabat); WSG1 (Abukarkatita); WSG2 (Abusabiba); WSG3 (Adar Abusabiba); WSG4 (Adar Umbatikh); WSG5 (Almahkara); WSG6 (Hamadyat); WSG7 (Hamadyat Sharateet); WSG8 (PQ434); WSG9 (Umbatikh (boda resist)); WSG10 (Zahana)

Figure 1 shows the crude protein content of the sorghum genotypes grains. The analysis of these genotypes showed significant (*p* < 0.05) genetic variation for the total protein content. It was varied between 10.2% and 14.6%. Among all tested sorghum genotypes, the highest percentage of total protein content was encountered in the grains of PQ‐434 (14.6%), whereas the lowest one was demonstrated in the grains of AG8 cultivar (10.3%). The wild sorghum genotypes PQ‐434 displayed significantly higher total protein contents compared to the corresponding released cultivar AG8 (Figure 1).

**Figure 2:**
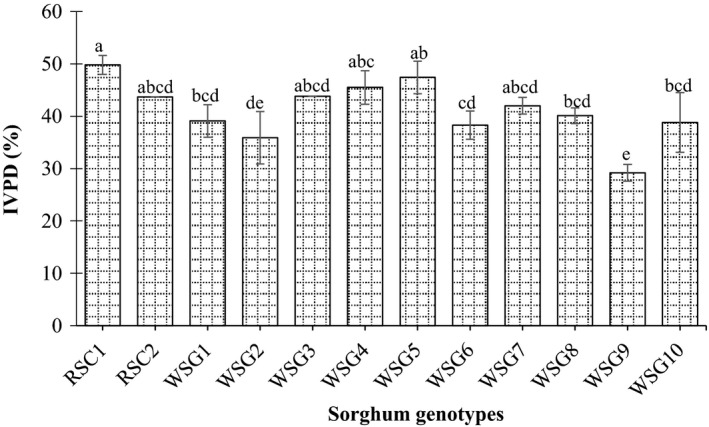
In vitro protein digestibility (%) of released sorghum cultivar and wild sorghum genotypes grains. Values are means (±*SD*) of triplicate samples. Values followed by the same letter are not significantly different (*p* < 0.05) as assessed by LSD. RSC1 (AG8); RSC2 (Tabat); WSG1 (Abukarkatita); WSG2 (Abusabiba); WSG3 (Adar Abusabiba); WSG4 (Adar Umbatikh); WSG5 (Almahkara); WSG6 (Hamadyat); WSG7 (Hamadyat Sharateet); WSG8 (PQ434); WSG9 (Umbatikh (boda resist)); WSG10 (Zahana)

In relation to the in vitro protein digestibility (IVPD), it should be noted that significant (*p* < 0.05) variation was observed among the wild sorghum genotypes and released cultivars. The maximum in vitro protein digestibility was determined in grains of the released cultivar AG8 (49.8%), followed by the wild genotype Almahkara (47.4%), whereas the lowest content was obtained in grains of the wild genotype Umbatikh boda resist (29.2%). The released sorghum cultivar AG8 showed significantly higher in vitro protein digestibility compared to those of wild sorghum genotypes Abusabiba, Hamadyat, PQ‐434, Umbatikh boda resist (29.2%), and Zahana. However, the wild sorghum genotypes Adar Abusabiba, Adar Umbatikh, Almahkara, and Hamadyat Sharateet displayed similar in vitro protein digestibility compared to that of the released cultivars AG8 and Tabat (Figure 2).

From obtained results, it was clearly observed that most of the wild sorghum genotypes grains contain relatively high protein content with high digestibility rate. Since, protein is an essential element of the food of animals and human and supplies the required amino acids (Okareh, Adeolu, & Adepoju, 2015). The crude protein of the wild sorghum genotypes might serve as an important source of new genetic material as well as fortified ingredients for enhancing the nutritional value of grains; subsequently, most of the studied genotypes contain higher protein content in comparison with Sudanese sorghum cultivars Gadambalia, Tabat, and Wad ahmed (Ahmed, Abdalla, Inoue, Ping, & Babiker, 2014; Elbashir, Mustafa, Tinay, & Babiker, 2008).

### 3.2 Total and available mineral of the wild sorghum genotypes and released sorghum cultivars

The total content and availability of Ca, P, Fe, and Zn of the wild sorghum genotypes and released sorghum cultivars are shown in Table 1. It was clearly observed that the total and bioavailability of the elements varied among the sorghum genotypes. However, the obtained data indicated that most of the studied genotypes grains are rich with the major element. On the other hand, significant (*p* < 0.05) variation in the total and available content was observed among the genotypes (Table 1).

**Table 1:** Total and available Ca, P, Fe, and Zn of selected released and Sudanese wild sorghum genotypes

Sorghum genotypes	Ca		P		Fe		Zn	
	Total (mg/g)	Available (%)	Total (mg/g)	Available (%)	Total (mg/100g)	Available (%)	Total (mg/100g)	Available (%)
RSC1	2.2 ± 0.40^b^	17.3 ± 2.28^bcd^	1.66 ± 0.23^a^	17.3 ± 2.3^abc^	1.18 ± 0.23^e^	61.2 ± 7.6^bc^	0.45 ± 0.02^e^	88.7 ± 0.6^a^
RSC2	1.5 ± 0.15^c^	20.9 ± 5.88^abc^	1.46 ± 0.01^a^	17.1 ± 4.2^abc^	1.28 ± 0.43^de^	75.7 ± 1.9^ab^	0.54 ± 0.01^de^	80.6 ± 9.6^a^
WSG1	2.4 ± 0.50^b^	9.3 ± 1.47^e^	1.45 ± 0.18^a^	9.3 ± 1.50^c^	1.32 ± 0.44^cde^	71.2 ± 8.2^abc^	0.56 ± 0.16^cde^	69.8 ± 8.6^abc^
WSG2	1.4 ± 0.20^c^	22.7 ± 0.74^ab^	1.51 ± 0.09^a^	22.7 ± 4.3^ab^	1.30 ± 0.33^cde^	92.8 ± 0.1^a^	0.61 ± 0.03^cde^	58.0 ± 9.9^bcd^
WSG3	0.7 ± 0.20^ef^	23.2 ± 4.06^ab^	1.70 ± 0.16^a^	23.2 ± 4.1^ab^	1.49 ± 0.34^bc^	52.0 ± 0.6^c^	0.65 ± 0.01^bcd^	50.4 ± 2.5^cde^
WSG4	0.7 ± 0.30^ef^	13.9 ± 2.21^de^	1.52 ± 0.24^a^	14.0 ± 3.8^bc^	1.61 ± 0.29^ab^	49.7 ± 9.8^c^	0.87 ± 0.03^a^	37.2 ± 2.2^e^
WSG5	1.3 ± 0.30^cd^	23.1 ± 0.26^ab^	1.92 ± 0.19^a^	19.5 ± 0.1^abc^	1.59 ± 0.09^ab^	61.9 ± 4.8^bc^	0.72 ± 0.01^abc^	75.0 ± 3.1^ab^
WSG6	1.1 ± 0.25^cde^	13.9 ± 2.21^de^	1.58 ± 0.06^a^	13.9 ± 2.2^bc^	1.40 ± 0.23^bcd^	68.4 ± 9.6^bc^	0.63 ± 0.04^cd^	46.1 ± 0.0^de^
WSG7	0.5 ± 0.10^f^	18.5 ± 1.12^bcd^	1.77 ± 0.20^a^	18.5 ± 5.1^abc^	1.64 ± 0.25^ab^	70.9 ± 1.5^bc^	0.7 ± 0.01^abcd^	50.4 ± 1.3^cde^
WSG8	0.9 ± 0.15^def^	14.9 ± 1.65^cde^	1.98 ± 0.35^a^	14.9 ± 2.3^bc^	1.34 ± 0.34^cde^	75.5 ± 0.3^ab^	0.81 ± 0.03^ab^	69.4 ± 0.0^abc^
WSG9	2.7 ± 0.30^a^	8.5 ± 0.23^e^	1.13 ± 0.42^a^	8.5 ± 0.20^c^	1.91 ± 0.22^a^	64.0 ± 7.7^bc^	0.63 ± 0.09^cd^	75.0 ± 1.3^ab^
WSG10	1.2 ± 0.15^cde^	27.8 ± 2.73^a^	1.51 ± 0.04^a^	27.8 ± 2.7^a^	1.20 ± 0.43^e^	78.5 ± 4.9^ab^	0.68 ± 0.05^bcd^	87.3 ± 2.2^a^
SE±	0.12	1.15	0.30	3.22	0.05	2.23	0.05	5.52
CV%	25.0	20.0	23.7	22.9	15.4	4.99	8.9	10.3

Note

Values are means (±) *SD* of triplicate samples. Values without letters are not significantly different (*p* < 0.05) as assessed by Tukey. RSC1 (AG8); RSC2 (Tabat); WSG1 (Abukarkatita); WSG2 (Abusabiba); WSG3 (Adar Abusabiba); WSG4 (Adar Umbatikh); WSG5 (Almahkara); WSG6 (Hamadyat); WSG7 (Hamadyat Sharateet); WSG8 (PQ‐434); WSG9 (Umbatikh (boda resist)); WSG10 (Zahana).

Across all wild sorghum genotypes, the total Ca content of the grains varied from 0.5 mg/g for Hamadyat Sharateet genotype to 2.7 mg/g for the Umbatikh (boda resist genotype), while it was found to be 2.2 and 1.5 mg/g for the released cultivars AG8 and Tabat, respectively. On the other hand, the bioavailability of Ca was also varied significantly (*p* < 0.05) among the genotypes. The wild genotypes Zahana, Almahkara, Adar Abusabiba, and Abukarkatita contain higher values of Ca availability compared to those of released sorghum cultivars AG8 and Tabat (Table 1).

Result presented in Table 1 shows that the P content is varied across the genotypes; however, no significant (*p* < 0.05) differences were observed in P content between the genotypes. The maximum concentrations of total phosphorus were found in grains of wild sorghum genotype PQ‐434 (1.98 mg/g) followed by Almahkara (1.92 mg/g), whereas the lowest concentration was obtained in grains of wild sorghum accession Umbatikh boda resist (1.13 mg/g). Across all sorghum genotypes, the bioavailable phosphorus contents of the grains varied from 8.5% for the wild sorghum accession Umbatikh boda resist to 27.8% for the wild sorghum genotype Zahana with an average of 17.2% (Table 2). The grains of wild sorghum genotype Zahana were found to contain significantly higher bioavailable phosphorus contents compared to those of wild sorghum accessions Abukarkatita, Adar Umbatikh, Hamadyat, PQ‐434, and Umbatikh boda resist. Nonetheless, the bioavailable phosphorus contents of wild sorghum genotypes Abusabiba and Adar Abusabiba were significantly higher than those of wild sorghum genotypes Abukarkatita and Umbatikh boda resist.

**Table 2:** Phenotypic correlation among mineral elements and protein contents of selected released and Sudanese wild sorghum genotypes

	Crude protein	IVPD	Total Ca	Available Ca	Total P	Available P	Total Fe	Available Fe	Total Zn	Available Zn	Tannin	Total polyphenol	Phytic acid
Crude protein	1												
IVPD	0.02	1											
Total Ca	0.56	−0.17	1										
Available Ca	0.45	0.35	−0.31	1									
Total P	0.56	0.57*	0.33	0.37	1								
Available P	0.37	0.28	−0.30	0.97***	0.36	1							
Total Fe	0.36	0.16	−0.13	0.07	0.30	−0.06	1						
Available Fe	−0.13	−0.52	0.21	−0.01	−0.32	0.09	−0.80**	1					
Total Zn	0.36	−0.02	0.22	0.00	0.40	0.03	0.31	−0.27	1				
Available Zn	0.35	0.04	0.26	0.21	−0.13	0. 12	−0.01	0.09	−0.52	1			
Tannin	−0.13	0.00	−0.46	0.28	−0.25	0.32	0.00	−0.23	0.40	−0.31	1		
Total polyphenol	−0.46	−0.44	−0.57	0.06	−0.52	0.14	0.09	0.05	0.10	−0.35	0.51	1	
Phytic acid	−0.37	−0.03	−0.22	−0.11	−0.01	−0.10	−0.17	0.27	0.12	−0.58	0.03	0.11	1

Values with *are significant at *p* < 0.05; **p < 0.01; ***p < 0.001.

Grain mineral analysis showed a significant (*p* < 0.05) genetic variation for total and available iron and zinc among the sorghum genotypes (Table 1). The total iron concentrations of the grains varied from 1.18 to 1.91 mg/100 g with overall mean of 1.25 mg/100 g. Across all sorghum genotypes, Umbatikh (boda resist) grains were found to possess significantly higher total iron concentration compared to the other genotypes. The highest availability values of iron were recorded in grains of the wild genotype Abusabiba (92.8%) followed by Zahana (78.5%). Grains of the wild genotype collected from Adar Umbatikh area were found to possess the lowest available iron concentration (49.7%). The wild sorghum genotype Abusabiba exhibited significantly higher available iron concentrations compared to that of the released sorghum cultivar AG8. The released sorghum cultivars demonstrated significantly higher available iron contents than those of wild sorghum genotypes Adar Umbatikh and Almahkara (Table 1).

The concentration of Zn in grains of different wild sorghum genotypes and released sorghum cultivars is shown in Table 1. Across all released sorghum cultivars, the wild sorghum Adar Umbatikh grains were found to possess higher total zinc contents. Grains of the wild sorghum accessions Adar Umbatikh, Almahkara, and PQ‐434 demonstrated significantly higher total zinc contents than those of the released sorghum cultivars. Similar to the total iron contents, grains of the released sorghum cultivar AG8 were found to possess the lowest total zinc concentration of 0.45 mg/100 g compared to wild genotypes. Zinc available in the grains was found to be varied from 37.2% for the wild sorghum Adar Umbatikh to 88.7% for the released cultivar AG8 with overall mean of 65.7%. Grains of the wild sorghum accessions Abukarkatita, Almahkara, PQ‐434, Umbatikh boda resist, and Zahana displayed almost similar available zinc values compared to the corresponding released cultivars. Similar to Fe, all studies wild genotypes are relatively containing high amount of Zn compared to other sorghum cultivars; however, most of them showed lower rate of Zn bioavailability.

Regarding to these findings, high concentration of Fe and Zn in the studied wild sorghum genotypes might offer a potential source for the biofortification through plant breeding program. Since biofortification is considered as the most suitable solution for the severe malnutrition problem particularly in the developing countries (Clemens, 2014; Stein, 2010).

Generally, our findings show that the wild sorghum genotypes have high concentration of macro‐ and microelements. However, a significant (*p* < 0.05) variation was observed among the genotypes. These variations might be due to the genetic variability between species and the effects of environment of the collection sites. The genetic variability for content of the elements could be admitted as a desired trait of Sudanese wild sorghums. It has been reported that among the blackgram genotypes, the genetic variability showed significant factor on the variation of Zn and Fe bioavailability (Singh, Kanaujia, Srivastava, Dixit, & Singh, 2017). Therefore, it is imperative to raise greater awareness for breeders to study the nutritional status of diverse Sudanese wild sorghums in order to find genotypes with elevated grain micronutrient concentrations as the initial step for a breeding program.

### 3.3 Antinutritional factor content of the wild sorghum genotypes and released sorghum cultivars

**Figure 3:**
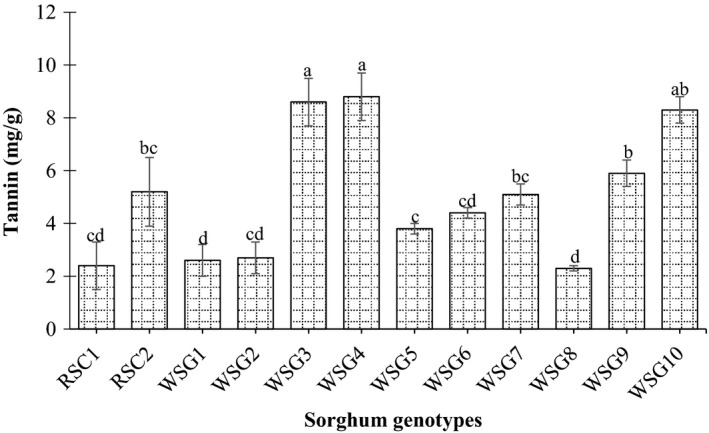
Tannin content (mg/g) of released sorghum cultivar and wild sorghum genotypes grains. Values are means (±*SD*) of triplicate samples. Values followed by the same letter are not significantly different (*p* < 0.05) as assessed by LSD. RSC1 (AG8); RSC2 (Tabat); WSG1 (Abukarkatita); WSG2 (Abusabiba); WSG3 (Adar Abusabiba); WSG4 (Adar Umbatikh); WSG5 (Almahkara); WSG6 (Hamadyat); WSG7 (Hamadyat Sharateet); WSG8 (PQ434); WSG9 (Umbatikh (boda resist)); WSG10 (Zahana)

In this study, the antinutritional factors in wild sorghum genotypes and released cultivars were determined in the term of phytic acid, tannin, and total polyphenol content. In general, the antinutritional factor contents of ten Sudanese wild sorghum accessions and two released cultivars showed a significant (*p* < 0.05) genetic variation for tannins, total polyphenols, and phytic acid. The grain tannin content ranged from 2.3 to 8.8 mg/g with an average of 5 mg/g. The wild sorghum accessions PQ‐434, Abukarkatita, and Abusabiba gave significantly lower grain tannin contents and were not statistically different compared to those of released sorghum cultivar AG8 (Figure 3). However, contents of tannins were higher in wild sorghum genotype Adar Umbatikh (8.8 mg/g) followed by Adar Abusabiba (8.6 mg/g) and were significantly different compared to that of the released cultivars AG8 and Tabat.

Tannins can form cross‐linkages between proteins and other macromolecules and render them unavailable for digestion (Griffiths, 1991). These inhibitory facets, in conjunction with an astringent taste, constitute the antinutritional characteristics of tannins (Petterson, 2000). The antinutritive activity of tannin is due to its ability to make complexes with protein as well as with digestive enzymes (Griffiiths 1999).

**Figure 4:**
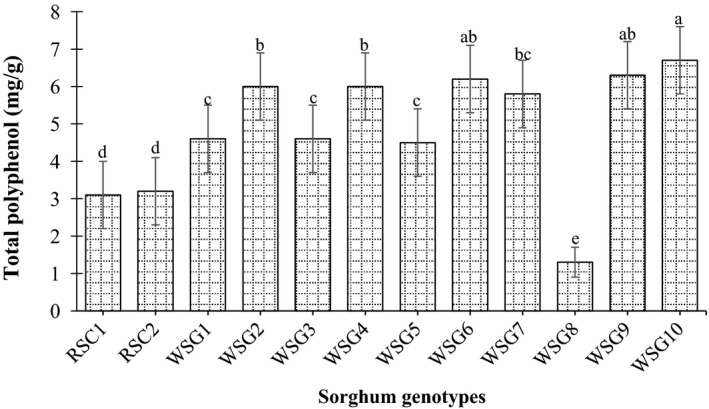
Total polyphenol (mg/g) of released sorghum cultivar and wild sorghum genotypes grains. Values are means (±*SD*) of triplicate samples. Values followed by the same letter are not significantly different (*p* < 0.05) as assessed by LSD. RSC1 (AG8); RSC2 (Tabat); WSG1 (Abukarkatita); WSG2 (Abusabiba); WSG3 (Adar Abusabiba); WSG4 (Adar Umbatikh); WSG5 (Almahkara); WSG6 (Hamadyat); WSG7 (Hamadyat Sharateet); WSG8 (PQ434); WSG9 (Umbatikh (boda resist)); WSG10 (Zahana)

The lowest total polyphenol contents were detected in grains of wild sorghum genotype PQ‐434 (1.3 mg/g), whereas the highest total polyphenol contents were recorded in grains of wild sorghum genotypes collected from Zahana (6.7 mg/g). However, the wild sorghum genotype PQ‐434 significantly contains the lower concentration of the total polyphenol than the released sorghum cultivars AG8 and Tabat (Figure 4). Low concentration of total polyphenols may also enhance the bioavailability of trace elements, since they are able to create linkage and form mineral complex which reduces its extractability. (Abdelrahman et al., 2007). Such variations could be attributed to genetics, physiological/biochemical mechanisms, responses to climate variations, tolerance to pests and diseases, and responses to agronomic management practices. Genetic variations in plant acquisition of nutrients have been reported (Duncan & Carrow, 1999).

**Figure 5:**
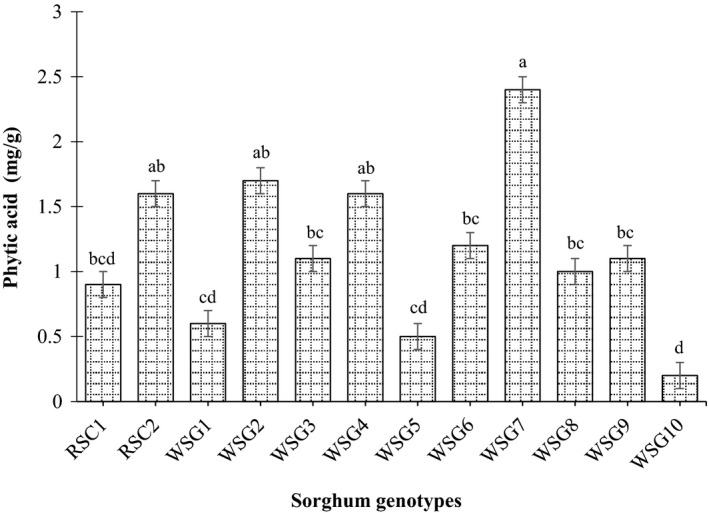
Phytic acid content (mg/g) of released sorghum cultivar and wild sorghum genotypes grains. Values are means (±*SD*) of triplicate samples. Values without letters are not significantly different (*p* < 0.05) as assessed by LSD. RSC1 (AG8); RSC2 (Tabat); WSG1 (Abukarkatita); WSG2 (Abusabiba); WSG3 (Adar Abusabiba); WSG4 (Adar Umbatikh); WSG5 (Almahkara); WSG6 (Hamadyat); WSG7 (Hamadyat Sharateet); WSG8 (PQ434); WSG9 (Umbatikh (boda resist)); WSG10 (Zahana)

For all sorghum genotypes, grains’ phytic acid contents varied from 0.2 mg/g for wild sorghum genotype Zahana to 2.4 mg/g for wild sorghum genotype Hamadyat Sharateet with an average of 1.1 mg/g (Figure 5), while the wild sorghum genotypes Zahana, Almahkara, and Abukarkatita had significantly lower phytic acid contents compared to the released sorghum cultivar Tabat (Figure 5). Phytic acid can complex protein as well as trace elements and lower their availability (Carnovale, Lugaro, & Lombardi‐Boccia, 1988). Hence, low phytic acid content in wild sorghum genotypes grains might result in high bioavailability rate of mineral particularly trace one.

In comparison with the antinutritional factors, tannin, total polyphenol, and phytic acid, in Sudanese sorghum cultivars Gadambalia, Tabat, and Wad ahmed wild genotypes grains, contain less amount of antinutrients than those of released sorghum cultivars (Ahmed et al., 2014; Idris, Hassan, Babiker, & Tinay, 2005). Moreover, the variation of the phytochemical compound among the wild sorghum genotypes might be due to several factors particularly the growing environment. It has been reported by Lu et al. (2015) that the environment played a larger role in variations of individual phytochemical component and antioxidant property of soft red winter wheat bran than did other factors.

### 3.4 HJ‐biplot analysis

**Figure 6:**
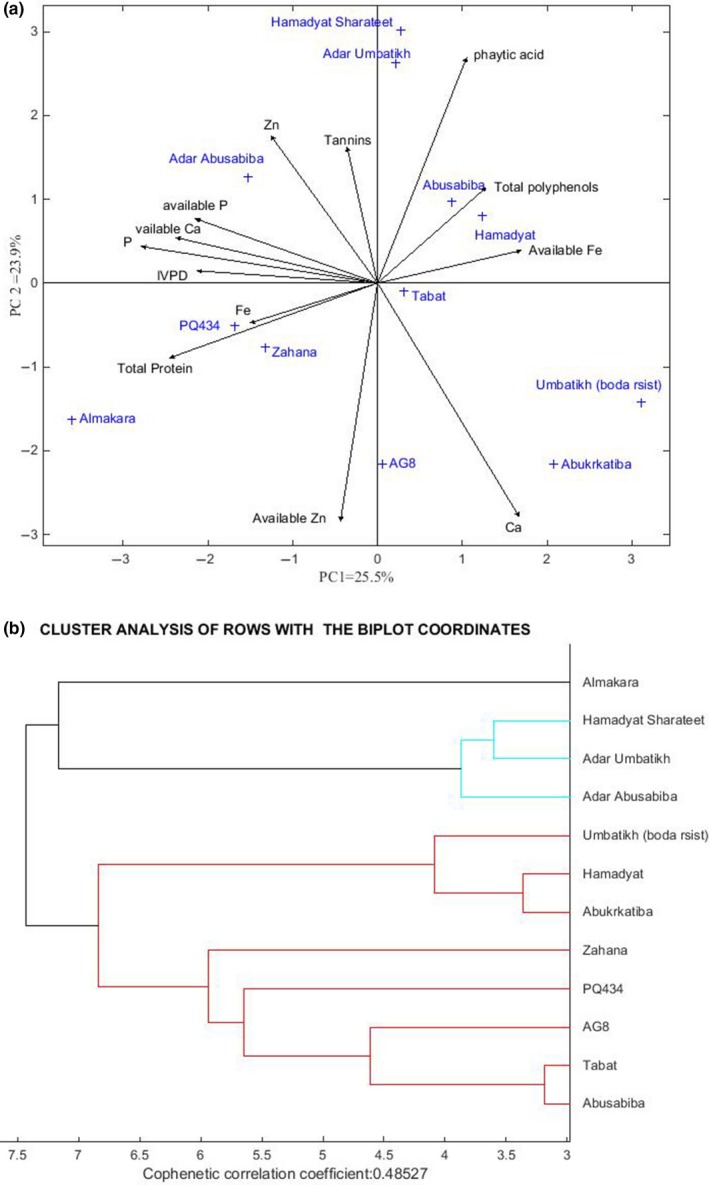
(a) Principle component analysis (PCA1 and PCA2 = 48%) of crude protein content, in vitro protein digestibility (IVPD), antinutritional factors, and mineral content of selected released and Sudanese wild sorghum genotypes. (b) Dendrogram showing the clustering pattern of 12 sorghum accessions from Sudan based on biplot coordinates.

Principal component analysis (PCA) is a technique used to emphasize variation among the genotypes and to identify patterns in the dataset used for the analysis. In this study, the HJ‐biplot analysis was conducted by comparing the principal component analysis (PCA) to strongly determine the multivariate interactions between the quality attributes together with the wild sorghum genotypes and released sorghum cultivars in order to identify patterns in the dataset used for the analysis table (Figure 6a,b). As illustrated in Figure 6a, the result of the first principal component analysis (PCA1) is explained as 25.9% of the total variability among the genotypes, whereas the second principal component analysis (PCA2) is reported as 23.9%. Hence, results of the PCA1 and PCA2 accounted for about 49.4% of the total variability representing a remarkable contribution of the two PC axes to the plotted components (Figure 6a). As reported by Yan and Fregeau‐Reid (2008), the eccentricity of characters that appear <90° angle is positively correlated, while the factors that formed more than 90° angles were associated with negative correlation, and those who have a 90° angle do not show a correlation in the biplot. Accordingly, the data revealed that the wild sorghum genotypes Almahkara, Hamadyat Sharateet, Adar Umbatikh, and Adar Abusabiba were commonly distant compared to the other genotypes. On the other hand, the released sorghum cultivar Tabat and wild sorghum genotypes Hamadyat and Abusabiba were mostly stable in terms of mineral and protein contents (Figure 6a).

Cluster analysis based on Euclidean distance was performed to employ associations among the traits for assigning the germplasm lines and varieties to different clusters. Genotypes were grouped based on biplot coordinates into three main clusters (Figure 6b). Cluster indicated that genotypes Tabat, Abusabiba, Hamadyat, Abukarkatita, Adar Umbatikh, and Hamadyat Sharateet revealed relatively similar mineral concentration. This indicated that they had some common characters in the uptake, translocation, and the biological processes of this mineral element. Genotype Almahkara was separated as a singleton accession from the rest in this cluster (Figure 6b). This accession consisted of high crude protein and IVPD. The study on nutritional contents variation has shown that identification of sorghum germplasm for breeding for improvement of mineral nutrients and protein is promising. High‐yielding germplasm lines with improved micronutrient levels were grouped together (Tabat and Abusabiba). Selection and crossing of genotypes from different clusters would help in bringing together genes favorable for yield and quality traits so as to breed tailor‐made varieties (Badigannavar, Girish, & Ganapathi, 2015; Shergo, Labuschagne, Shargie, & van Biljon, 2013).

### 3.5 Phenotypic correlation among nutrients

Correlation coefficients among mineral elements and protein are given in Table 2. The correlation among protein and the total and available Fe showed a negative and highly significant correlations (*R* = −0.80**). Availability of Zn and Ca was significant and positively correlated (*R* = 0.59*); Fe showed positive but not significantly correlated with Zn and total protein (Table 2). Significant correlation between Fe, Zn, and protein was observed in most of the studies (Wang, Yin, Tanaka, Tanaka, & Tsujimoto, 2011). This has implicated for the possibility of combining selection for these micronutrients in a single agronomic background. Genetic mapping in different wheat populations confirmed QTL colocalization conferring high protein, high Zn, and high Fe (Peleg et al., 2009). Likewise, colocalization of QTLs for Zn and Fe concentrations has been reported in rice (Garcia‐Oliveira, Tan, Fu, & Sun, 2009). Some genes like grain protein content‐B1 gene (Tt‐NAM‐B1; Gpc‐B1), which is located on chromosome 6BS, can increase the Fe and Zn translocation from leaves to seeds resulting in increased Fe and Zn accumulation in seeds (Wang et al., 2011). Similar relationship between protein, Fe, and Zn has been reported in sorghum (Ng'uni, Geleta, Hofvander, Fatih, & Bryngelsson, 2012) and rice (Garcia‐Oliveira et al., 2009). IVPD was positively and significantly correlated with P (*R* = 0.61*) and the availability of P observed to be totally linked with Ca availability (*R* = 0.96***). Highly positive significant correlation between IVPD and P revealed that concurrently improvement of these nutrients is possible. Correlations between traits are of great importance for the success of selection practiced in breeding programs. Zn content was positively correlated with Ca, suggesting the possibility of combining selection for both micronutrients in a single agronomic background. The association between traits and their contribution to diversity can be validated by multivariate analysis. Breeding for high total protein content in such a manner increases the concentration of Mn, P, and Zn and vice versa. Waters and Pedersen (2009) also reported positive correlation between sorghum grain protein and Zn and P.

### 3.6 Estimation of broad‐sense heritability

Broad‐sense heritability (H2) of the mineral elements and protein was estimated from the analysis of variance following Nyquist and Baker (1991). Estimates of broad‐sense heritability (H2) exhibited medium to high % and ranged from 97% for P to 40% for Fe (Table 3). Lower level of estimated broad‐sense heritability indicated the strong environmental effect and proportion of phenotypic variance attributable to environmental variance to genotype variance especially for Fe and P (Table 3). Total phenol, total protein, Ca, and P were strongly affected by the environmental factors compared to the rest of the nutrients (Table 3). Low H2 values for some traits indicated that there are no specific adaptation patterns for the accessions studied, so when breeding for higher nutrient content of these minerals in sorghum, any of the locations may be used for cultivation. Heritability estimates are limited to experimental material and setup and may differ widely in the same crop and same trait (Garcia‐Oliveira et al., 2009). Heritability is a measure of genetic differences among individuals in a population, not simply of whether or not a trait is inherited (Gomez‐Becerra et al., 2010). As decribed in Table 3, tannin, phytic acid, available Fe, Fe, available Zn, Zn appeared to be highly variable trait with high values of heritability more than 85%.

**Table 3:** Broad‐sense heritability (H^2^), chi‐square test (χ^2^), and Chi probability of 14 measured variables of selected released and Sudanese wild sorghum genotypes

Traits	H^2^	Chi (χ^2^) probability	Chi‐square (χ^2^) test
Crude protein	0.73	0.0072	7.22
IVPD	0.91	0.000	22.70
Total Ca	0.53	0.137	2.20
Available Ca	0.80	0.0009	11.11
Total P	0.40	0.300	1.03
Available P	0.83	0.0003	13.23
Total Fe	0.97	0.0000	39.92
Available Fe	0.93	0.0000	27.47
Total Zn	0.92	0.0000	25.54
Available Zn	0.95	0.0000	32.23
Tannin	0.94	0.0000	30.44
Total phenol	0.53	0.1301	2.29
Phytic acid	0.92	0.0000	23.60

## 4 CONCLUSION

Generally, obtained results revealed that there is genetic variation among genotypes. These variations might help to produce sorghum cultivars with high nutritional quality through breeding in the available gene tool. The results of our experiment showed that the wild sorghum accession PQ‐434 exhibited fairly low tannins, total polyphenols, and high crude protein contents compared to the released sorghum cultivars, with a considerable potential for improvement of the domesticated Sudanese sorghum. On the other hand, S. bicolor var arundinaceum collected from Almahkara and PQ‐434 are to be considered as potential sources for iron and protein biofortification, respectively. These identified sources can be utilized in wide hybridization for transferring useful genes from alien species into cultivated sorghum. Moreover, to diversify and broaden the genetic base of cultivated sorghum, introgression of alien genes from wild sorghum needs to be persuaded vigorously to make discernible yield advances in sorghum. In general, high correlation value of wild sorghum genotypes to released sorghum cultivars shows that these genotypes are potentially excellent materials to the sorghum breeders to produce high quality of sorghum cultivars. Therefore, concerted efforts are required to screen more wild resources of sorghum for minerals and other nutritional traits.

## CONFLICT OF INTEREST

Authors declare that there is no conflict of interests.

## ETHICAL APPROVAL

This work does not involve any human or animal experiments.
